# Effect of xylitol on cariogenic and beneficial oral streptococci: a randomized, double-blind crossover trial

**Published:** 2012-06

**Authors:** A Bahador, S Lesan, N Kashi

**Affiliations:** 1 Department of Microbiology, School of Medicine, Tehran University of Medical Sciences, Tehran, Iran; 2 Department of Oral Medicine and Diagnosis, Dental Branch, Islamic Azad University, Tehran, Iran

**Keywords:** Cariogenic agents, Chewing gum, Sorbitol, *Streptococcus*, Xylitol

## Abstract

**Background/purpose:**

Although habitual consumption of xylitol reduces cariogenic streptococci levels, its effect on beneficial oral streptococci is less clear. The main aim of the study is to investigate the effect of short-term xylitol consumption on the oral beneficial streptococci level of saliva, *Streptococcus sanguinis* and *S. mitis*.

**Material and Methods:**

Twenty four volunteers with a median age of 23.7 years (range: 20-28) harboring *Streptococcus mutans, S. sobrinus, S. sanguinis* and *S. mitis* participated in the randomized, double-blind, cross-over study. The experimental chewing gum (1.5 g/pellet) contained 70% xylitol w/w while the control gum contained 63% sorbitol w/w. Saliva samples were collected before and after two three-week test periods with a four-week washout interval. Colony-forming units (CFU)/ml were enumerated for the estimation of *S. mutans* levels on Mitis Salivarius-Mutans valinomycin (MS-MUTV), *S. sobrinus* on Mitis Salivarius-Sobrinus (MS-SOB), *S. sanguinis* on Modified Medium 10-Sucrose (MM10-S) and *S. mitis* on Mitis Salivarius Agar with Tellurite (MSAT) media.

**Results:**

The *S. mutans* and *S. sobrinus* counts of the saliva samples decreased significantly (p = 0.01 and p = 0.011, respectively) in the xylitol gum group but not in the sorbitol gum group. The salivary *S. sanguinis* and *S. mitis* counts did not decrease in both xylitol and sorbitol gum groups.

**Conclusions:**

Based on the findings of this study, xylitol consumption reduced *S. mutans* and *S. sobrinus* counts in saliva but appeared not to effect numbers of *S. sanguinis* and *S. mitis* in saliva. So, habitual consumption of xylitol reduces cariogenic streptococci levels without any effect on beneficial sterptococci for the oral cavity.

## INTRODUCTION

Extensive epidemiological evidence has established a positive correlation between mutans streptococci (MS), most notably *Streptococcus mutans* and *Streptococcus sobrinus* and caries (1, 2). The coexistence of *S. mutans* and *S. sobrinus* in dental biofilm and saliva is associated with higher caries experience than if only *S. mutans* is detected (3, 4). *Streptococcus sobrinus* seems to be capable of producing more acid than *S. mutans*. Thus, *S. sobrinus* existence represents an important additional risk factor for caries due to its potential to exacerbate caries activity. As children grow up, the proportion of children positive for *S. sobrinus* may increase (5).

*Streptococcus sanguinis*, one of the major species of the indigenous oral biota colonizing dental plaque, is usually associated with tooth surfaces free of caries. The ratio of *S. mutans* to *S. sanguinis* might be indicative of risk for caries or caries outcome. *Streptococcus sanguinis* may play an antagonistic or protective role against *S. mutans* colonization and it is associated with healthy periodontium. Thus, the colonization of certain oral streptococci such as *S. sanguinis* might be one factor offering protection against periodontitis (6, 7).

*Streptococcus mitis* plays an ecological role in the oral cavity. *Streptococcus mitis* releases rhamnolipidlike biosurfactants, which interferes with adhesion of cariogenic MS strain. Biosurfactants effectively stimulate detachment of MS from exposed surfaces or in a salivary conditioning film by the dynamic trim forces that occur in the oral cavity (8).

Xylitol is a polyol sweetener, which is not fermented by oral bacteria. Xylitol practically neutralizes low pH-values in the oral cavity with beneficial effects on oral health. Regular xylitol consumption, at enough doses reduces MS level in both plaque and saliva (9–12). *Streptococcus mutans* takes xylitol into the cell via a fructose phosphotransferase system (PTS) and xylitol is metabolized to xylitol-5-phosphate, which cannot be utilized further and may even be toxic to bacteria (11). Since we found fructose-PTS genes using NCBI resources in *S. sanguinis* and *S. mitis* genomes as well as *S. mutans*, the hypothesis of the study would be, although xylitol consumption would result in prevention of caries by decreasing the numbers of cariogenic agent, *S. sobrinus*, decreasing the beneficial oral bacteria such as *S. sanguinis* and *S. mitis* in the period of using xylitol chewing gum would initiate the oral health endangerment such as periodontitis.

Actually little is known about the clinical trial effects of xylitol and sorbitol on the caries-protective bacteria. Since several studies (9–12) have shown the effects of xylitol on *S. mutans* levels in saliva, we use *S. mutans* as internal control in this study. Considering no cross-over randomized study on the effect of xylitol and sorbitol on *S. sobrinus*, *S. sanguinis* and *S. mitis*, the aim of the present study is to evaluate the effect of xylitol on *S. sobrinus, S. sanguinis* and *S. mitis*.

## MATERIALS AND METHODS

### Subjects

Between Dec 2009 and Jun 2010 twenty-four healthy dental students from The Islamic Azad University-Dental Branch (Tehran, Iran) were participated in the study. The inclusion and exclusion criteria were as reported in Table 1. The included subjects were chosen on the basis of a pre-screening for the presence of *S. mutans*, *S. sobrinus*, *S. sanguinis* and *S. mitis*.

**Table 1:** Inclusion and exclusion criteria for participation in this study.

Inclusion criteria	Exclusion criteria
- Subject giving his written informed consent	-Systemic disease
- Subject considered as normal after clinical examination	-Infectious or inflammatory diseases in the last month
- Subject willing to comply with the study procedures	-Taking medicine: antibiotics or fluoride in the last month
	-Habitual consumers of xylitol products and sorbitol-containing products.
	- Mouth rinses
	-Abnormal salivary flow rates of paraffin-stimulated saliva (<1 ml/min)
	-Pregnancy
	-Contraceptive pill
	-Missed and filled tooth (MDFT) one filled and/or decayed (in dentine)
	-Abnormal or particular dietary habits
	-Consumption of food complements: antioxidant, …

### Experimental and Control Chewing Gums

The experimental chewing gum (1.5 g/pellet) contained 70% xylitol w/w (Orion Food Vina Co., Ltd. Binh Duong, Vietnam). The control gum was as the same as xylitol gum in pellet but the main sweetener was sorbitol, with a concentration of 63%. Gum containing sorbitol (Shantou Slg Foods Co., Ltd. Shantou, Mainland), was included in this study because it is also commonly used as a sweetener in many chewing gums; in fact, being less expensive, sorbitol is more frequently used than xylitol (13). They were packed in identical plastic containers which were colour-coded, yellow and red. The codes of the test gums were revealed after the results had been fed into SPSS files.

### Study Design

**Fig. 1:**
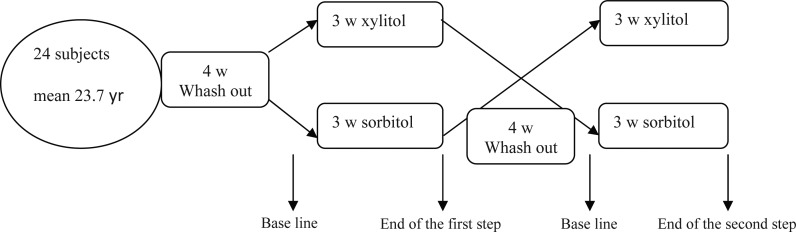
Algorithm of the study design.Yr, year; W, week.

The investigation had a cross-over randomized double-blind prospective design with two arms (14) (Fig. 1) that was approved by The Islamic Azad University Teaching and Research Ethics Committee (IAUTREC), Tehran, Iran. The study and intervention involved were completely explained to all participants, and written consent was obtained from all subjects. Following screening, subjects were randomly allocated into one of two groups. Subjects entered a four-week wash-out period followed by a three-week treatment period (Group AB/ part 1). At the end of this period there was a four week wash-out period. Subjects then entered a second three-week treatment period when they received the alternative treatment (Group BA/ part 2). Group AB consisted of two xylitol chewing gum pieces at a time, three times a day, after meals for three-weeks followed by four weeks wash out then matched control gum as the same as xylitol gum. Group BA consisted of control gum for three-weeks, four week washout, and then xylitol gum for three-weeks. The recommended chewing time was 10 min. The gum consumption resulted in a daily xylitol dose of approximately 6.6 g. The gum is not used during the washout periods.

The pre-experimental and post-experimental saliva were collected into sterile Flat-top cap for BD Falcon™ 50 ml conical centrifuge tubes (Becton, Dickinson; Tokyo, Japan). During this time the subjects were educated not to use mouthwashes, antibiotics or xylitol products, but to continue their usual tooth brushing and consume their normal diet. The subjects were instructed not to use tooth brushing and other oral hygiene procedures for 24 hours before sample collection and not to eat or drink for 1 hour before the sampling. At the appointments the subjects were also interviewed about confounding factors like acute infectious diseases and use of antibiotics. The subjects and researchers in this study were blinded throughout the study. Also the microbiological analyses in Tehran University of Medical Sciences (TUMS) were carried out blinded.

### Microbiological Analysis

The saliva samples were homogenized by ultra-sonication under ice-cold water for 10 s; 20 µl aliquots of 10-fold diluted samples were plated on the following selective media: Mitis Salivarius-Mutans valinomycin (MS-MUTV), Mitis Salivarius-sobrinus (MS-SOB), Modified Medium 10-Sucrose (MM10-S) and Mitis Salivarius Agar with Tellurite (MSAT) (QUELAB, UK). MS-MUTV medium is useful for the isolation of *S. mutans* alone from clinical samples (15). The usage of MS-SOB medium resulted in growth inhibition of *S. mutans* and oral streptococci other than *S. sobrinus* (16). *Streptococcus sanguinis* were selected from MM10-sucrose agar (17) based on their firm, adherent, star-shaped colony morphology. Growth of *S. mitis* on the MSAT agar appears as small or minute blue colonies (18). After 72 h of incubation at 37 °C in an anaerobic atmosphere, colony-forming units (CFU) were enumerated for the estimation of *S. mutans* levels on MS-MUTV, *S*. *sobrinus* on MS-SOB medium, *S. sanguinis* on MM10-S, *S. mitis* on MSAT media. For confirmation of the selectivity of media, colonies were identified biochemically using a rapid ID 32 STREP system (bioMérieux, France).

### Statistical Analysis

The data concerning *S. mutans*, *S. sobrinus*, *S. sanguinis* and *S. mitis* salivary levels at the four sampling phases were analyzed for a normal distribution. Differences between groups were assessed using the ANOVA test. The level of statistical significance was set at p < 0.05. The statistical software package used was SPSS 14.0 (SPSS Inc., Chicago, Ill., USA). For statistical analyses, where no bacterium detected, the levels of detection limit were 50 CFU/ml for each bacterial species (7).

## RESULTS

**Fig. 2:**
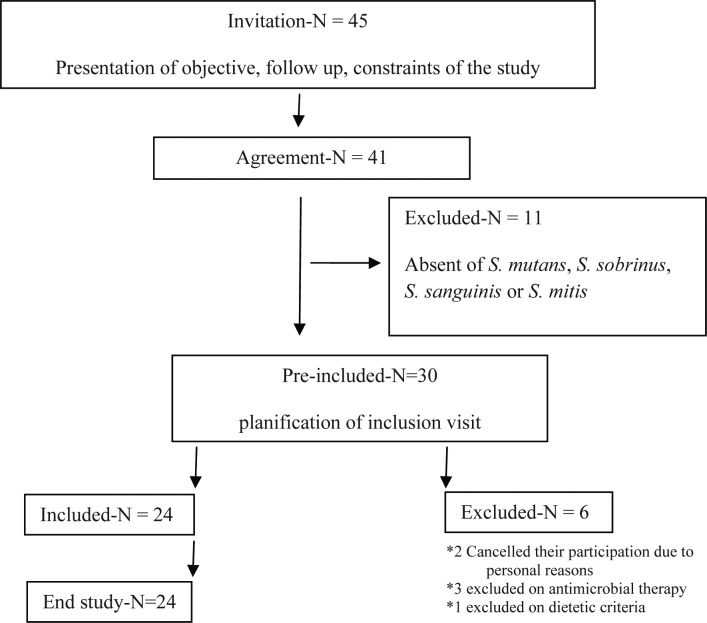
Flowchart of the subjects in the study.

Twenty-four (18 female and 6 male) of 30 Pre-included subjects, with a median age of 23.7 years (range: 20-28) completed the study. Two subjects cancelled their participation due to personal reasons, 3 persons excluded on antimicrobial therapy and 1 excluded on dietetic criteria (Fig. 2).

Since bacterial colony forming unit (CFU)/ml did not exhibit a normal distribution, the data were transformed to logarithms to confer homogeneity among the groups and then submitted to variance analysis with repeated measures. The original logarithmic values of CFU/ml data in Table 2 showed that there were very high variables of counts, particular in *S. mitis*, although all the xylitol groups indicated higher inhibitory tendency. All of studied subjects showed a reduction in *S. mutans* and *S. sobrinus* salivary levels in relation to baseline data. As shown in Table 2, the average *S. mutans* and *S. sobrinus* salivary levels were 3.38 and 2.88 (log_10_ CFU/ml) at baseline, respectively. After the experimental period the average levels of *S. mutans* and *S. sobrinus* in saliva decreased to 2.47 and 2.15 (log_10_ CFU/ml), respectively. In *S. mutans* salivary levels, the mean percentage of logarithmic value in the xylitol group dropped to 73% after 3 weeks, and this difference was statistically significant (P=0.01) in comparison to those in the control/sorbitol gum group. The mean percentage difference of logarithmic value for *S. mutans* between the xylitol gum group and the sorbitol gum group was slightly larger than the percentages for *S. sobrinus* (75%). The mean percent reduction of logarithmic values amongst *S. mutans* and *S. sobrinus* by xylitol were 27% and 21%, versus baseline, respectively. Variance analysis using ANOVA test revealed that the decrease in *S. mutans* and *S. sobrinus* levels observed in subjects after the xylitol chewing gum–phase and baseline was statistically significant (p = 0.01 and p = 0.011, respectively).

**Table 2:** The effect of xylitol vs. sorbitol chewing gum on the cariogenic and beneficial oral streptococci.

Microorganisms	Xylitol chewing gum			Sorbitol chewing gum		

	Baseline	After gum use	Mean percent reduction	Baseline	After gum use	Mean percent reduction
*S. mutans*			†			
Mean	3.38	2.47	27	3.22	3.12	3
SD	0.55	0.70		0.55	0.40	
Minimum	2.39	1.69		2.30	2.47	
Maximum	4.46	3.96		4.17	4.06	
Variance	0.30	0.49		0.23	0.16	
*S. sobrinus*			‡			
Mean	2.88	2.15	75	2.91	2.68	8
SD	0.53	0.65		0.41	0.49	
Minimum	2.00	1.69		2.00	1.69	
Maximum	4.11	3.94		3.88	3.64	
Variance	0.28	0.46		0.17	0.24	
*S. sanguinis*						
Mean	5.02	4.97	2	5.00	4.91	2
SD	0.30	0.40		0.27	0.26	
Minimum	4.36	4.14		4.41	4.45	
Maximum	5.64	5.97		5.49	5.32	
Variance	0.09	0.16		0.07	0.06	
*s. mitis*						
Mean	4.35	4.23	3	4.25	4.34	−2
SD	0.88	1.07		0.60	0.86	
Minimum	2.54	1.69		2.84	2.74	
Maximum	5.81	6.63		5.65	5.68	
Variance	0.79	1.16		0.66	0.74	

The counts (log_10_ CFU/ml) of each microorganism per saliva sample are expressed as logarithmic Values of mean±SD, Minimum, Maximum and Variance.

† It denotes a statistically significant difference p = 0.01

‡ It denotes a statistically significant difference p = 0.011.

No significant changes were seen in the beneficial bacteria counts of the xylitol gum group in comparison to those in the control/sorbitol gum group.

. Statistical analysis of the saliva samples from subjects after the control chewing gum–phase and baseline did not demonstrate significant differences amongst the different groups of bacteria (p > 0.05).

## DISSCUSION

This study is part of a series to explore the selective xylitol activity that can be used as anti-cariogenic agent. Although xylitol chewing gum has been reported to significantly reduce the mutans streptococci (MS) levels in plaque and saliva (9–12); its effect against *S. sobrinus* in a clinical trial has not been explored. Another pilot study in this series assessed the effect of xylitol on the composition of the oral flora. The results revealed xylitol consumption reduced MS counts in plaque but appeared not to affect the total microbial composition of plaque or saliva (7). In the present study, we tested the effectiveness of xylitol and sorbitol chewing gums in reducing load of *S. mutans* and *S. sobrinus*, as cariogenic agents and *S. sanguinis* and *S. mitis*, as oral health agents. The results showed that 3 weeks of xylitol chewing gum consumption reduced the levels of *S. mutans* and *S. sobrinus* in saliva compared to baseline levels and did not affect the *S. sanguinis* and *S. mitis* level. This supports the findings of previous studies proving xylitol reduces MS (10, 11).

*In vitro* studies have demonstrated that MS are target organisms of xylitol (19, 20). The inhibition effect of xylitol varies among MS strains (21, 22). It is reported that the ability of xylitol to act as an anticariogenic agent is most likely due to its ability to be transported into caries-causing oral bacteria, inducible fructose phosphotransferase system (PTS) and inhibiting fermentation either by depleting the cell of high-energy phosphate or by poisoning the glycolytic system (23, 24). Since fructose-PTS genes are detected in *S. mitis* and *S. sanguinis* as well as *S. mutans* and *S. sobrinus* using GenBank database, and their resistance to anticariogenic property of xylitol, it is suggested that there may be other unknown mechanisms of the xylitol effect against *S. mutans* and *S. sobrinus*. Söderling et al. (9) demonstrated that xylitol consumption did not reduce counts of either total salivary streptococci or streptococcal species determined with the DNA-DNA hybridization technique, *S. oralis*, *S. gordonii*, S*. salivarius* or *S. sanguinis*. Several oral lactobacilli also possess the fructose PTS pathway and thus could be inhibited by xylitol (25–27). The idea that xylitol consumption reduces counts of oral lactobacilli is controversial. Loesche et al. (28) found no effects induced by xylitol consumption on oral lactobacilli counts, but Mäkinen et al. (29) in the clinical trial demonstrated a xylitol-associated decrease in salivary lactobacilli. This result may, however, reflect a xylitol-induced elevation of the oral pH and therefore an indirect effect on salivary lactobacilli.

It was shown *in vitro* that xylitol could reduce adherence of *S. mutans* contributing to plaque and biofilm formation and induced changes in the virulence with an approach not dependent on growth inhibition (22, 30). In contradiction to the *in vitro* study of Sahni et al. (24), the present study showed that xylitol did not inhibit *in vivo S. sanguinis* in saliva*. In vitro* tests demonstrated the xylitol concentration of 12.5% was required for inhibiting the growth of *S. sanguinis*; however, *S. mutans* was inhibited significantly at a xylitol concentration of 1.56% (24). Kontiokari et al. (31) showed *in vitro* effectiveness of xylitol in reducing *S. mitis*. This is incompatible to the finding of the present study, which suggested that xylitol chewing gum consumption did not effect on *in vivo* growth of *S. mitis*. It is possible that xylitol selectively affects and reduces *S. mutans* and *S. sobrinus* levels without altering load of *S. mitis* and *S. sanguinis*, the bacteria implicated in development of oral health.

Aside from xylitol, studies involving sugar alcohols, most commonly sorbitol, suggests that they have little effect on actively reducing MS levels (32). This is consistant with our study, which showed no *in vivo* growth inhibition of sorbitol control gum for tested bacteria. So, it has suggested that the observed caries reduction through the sorbitol chewing process can be ascribed to stimulation saliva flow, increase in plaque pH, lack of sucrose and the inability of bacteria to metabolize polyols into acids instead of effect on reduction cariogenic bacterial level (33). Limitations of this study are as follow: diet uptake by each individual, small sample sizes and bias due to the nature of community intervention studies.

In conclusion, the results of the present study support the idea that *S. mutans* and *S. sobrinus* represent main target organisms of xylitol. It showes that consumption of xylitol in chewing gum reduces *S. mutans* and *S. sobrinus* in saliva but has no effect counts of *S. sanguinis* and *S. mitis*. Thus, we advocate that xylitol chewing gum consumption to maintain healthy ecology of the oral cavity.
